# A broad complex tachycardia suggesting global ischemia or repolarization abnormalities

**DOI:** 10.1002/joa3.12561

**Published:** 2021-06-02

**Authors:** Charis Gkalapis, Marios Papadakis, Claire A. Martin, George Bazoukis, Konstantinos P. Letsas, Konstantinos Vlachos

**Affiliations:** ^1^ Department of Electrophysiology‐Cardiology Klinikum Vest Recklinghausen Germany; ^2^ Second Surgical Department University of Witten Herdecke Wuppertal Germany; ^3^ Royal Papworth Hospital NHS Foundation Trust Cambridge UK; ^4^ Laboratory of Cardiac Electrophysiology "Evangelismos" General Hospital of Athens Athens Greece

**Keywords:** atrial flutter, shark fin phenomenon, ST segment elevation

## Abstract

Our case presents a ​cavotricuspid isthmus (CTI​)‐dependent atrial flutter (AFL) with 1:1 atrioventricular conduction and pronounced ST‐segment elevation in aVR, previously described as a “shark fin phenomenon,” mimicking a broad complex tachycardia with a ventricular origin.

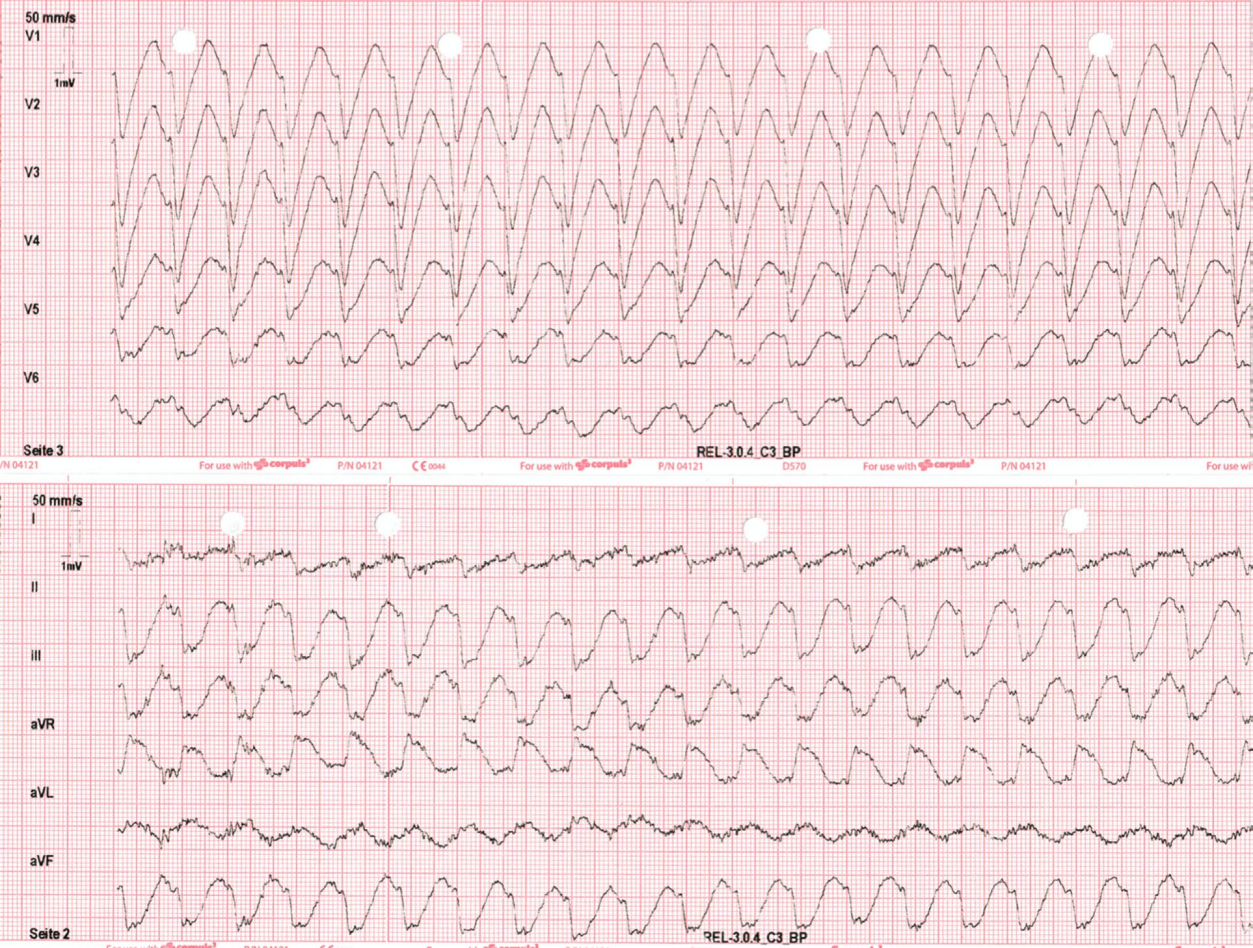

## 1 CASE REPORT

**FIGURE 1:**
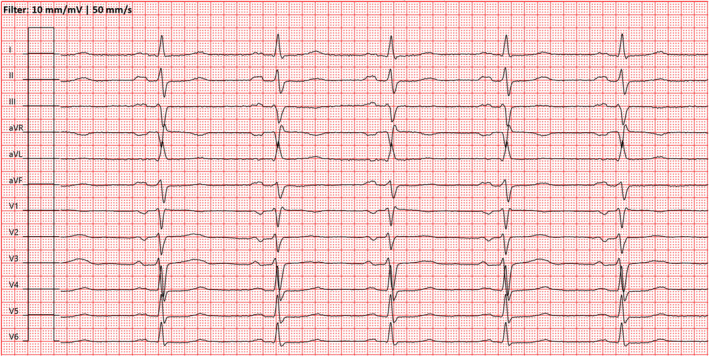
Two weeks before admission to the emergency department, the patient underwent successful ablation of an orthodromic AVRT due to a left lateral accessory pathway. Baseline ECG at discharge was normal without ST‐segment changes

A 59‐year‐old woman was admitted to the emergency department with palpitations due to tachycardia accompanied by chest pain and hypotension (80/60 mm Hg). Two weeks ago, the patient had undergone successful ablation of an orthodromic atrioventricular re‐entrant tachycardia (AVRT) due to a left lateral accessory pathway. Baseline ECG at discharge was normal without ST‐segment changes (Figure 1).

**FIGURE 2:**
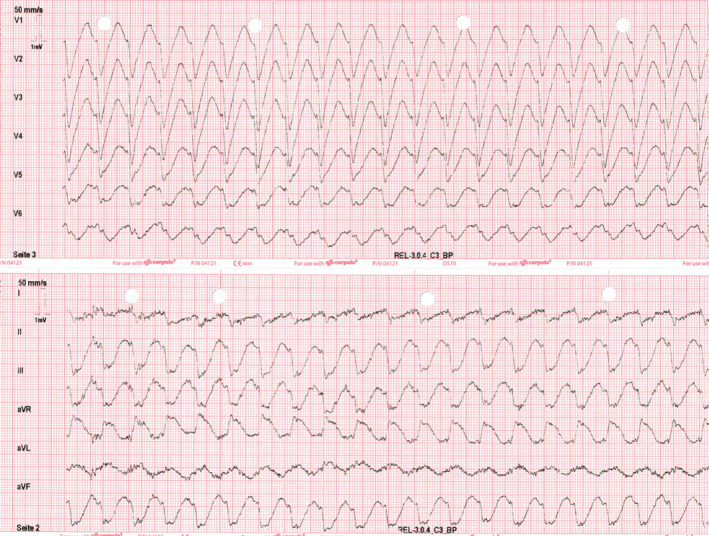
The patient presented to the emergency department with a broad complex tachycardia with a ventricular rate of 250 beats per minute. CTI‐dependent AFL with spontaneous 1:1 atrioventricular conduction and pronounced ST‐segment elevation in aVR, previously described as a “shark fin phenomenon”

**FIGURE 3:**
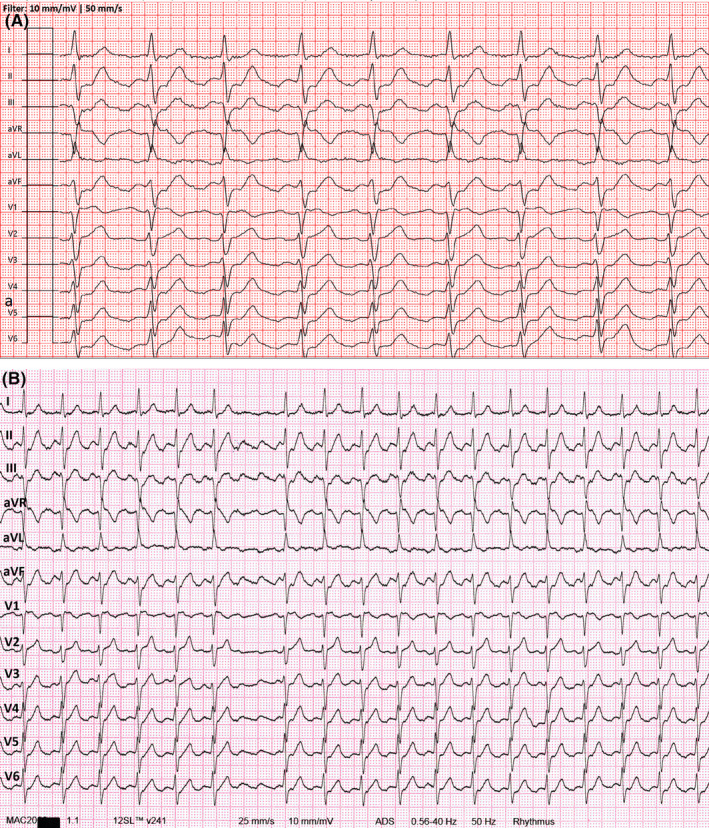
(A) After intravenous administration of 150 mg amiodarone, the ventricular rate decreased to 120 bpm with partial regression of ST changes. Negative P waves in inferior leads, positive P waves in V1, V2, and negative P waves V3‐V6 led to the suspicion of an atrial arrhythmia. (B) An adenosine test revealed CTI dependent atrial flutter with 2:1 ventricular conduction. A complete normalisation of ST changes with a slower ventricular response was observed. At that time, the patient was free of symptoms

The ECG on presentation demonstrated a broad complex tachycardia with a ventricular rate of 250 beats per minute and ST‐segment elevation (Figure 2). After intravenous administration of 150 mg amiodarone, the ventricular rate decreased to 120 bpm with partial regression of ST changes (Figure 3A).

### 1.1 What is your diagnosis?

### 1.2 Answer

The presence of negative P waves in the inferior leads, positive P waves in V1/V2, and negative P waves in V3‐V6 led to the suspicion of an atrial arrhythmia. We, therefore, performed an adenosine test, which revealed ECG appearance consistent with a cavotricuspid isthmus (CTI)‐dependent atrial flutter (AFL) (Figure 3B). Shortly after this, the ventricular response slowed further with almost complete resolution of the ST changes, as well as patient symptoms.

A transthoracic echocardiogram at a ventricular rate of 120 bpm revealed normal left ventricular function with an ejection fraction of 55% and without segmental hypokinesia or dyskinesia. Within 2 hours, high‐sensitive troponin (T‐hs) levels had increased from 238 U/L (normal range ≤167 U/L) to 535 U/L, indicating acute myocardial injury. Potassium and magnesium levels were within the normal range.

**FIGURE 4:**
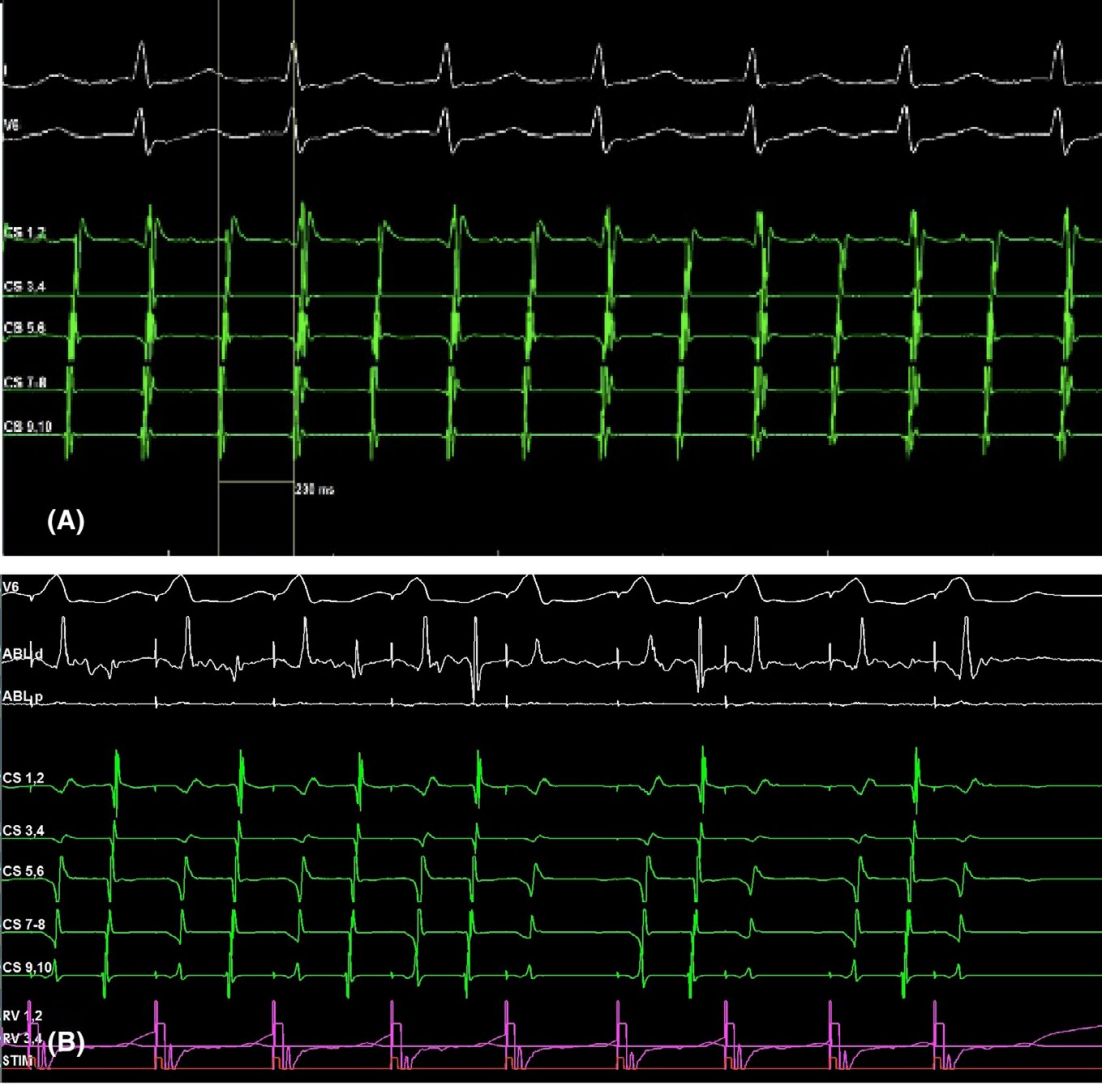
(A) An electrophysiological study confirmed the diagnosis of a typical counterclockwise flutter and sinus rhythm was restored after ablation on the cavotricuspidal isthmus achieving a bidirectional conduction block. (B) We confirmed the bidirectional block of the accessory pathway. RV pacing showed nodal retrograde VA activation

Coronary angiography excluded coronary artery disease involving the epicardial coronary vessels as the cause of the ischemia and ST changes. An electrophysiological study confirmed the diagnosis of typical counterclockwise AFL, and sinus rhythm was restored after ablation on the CTI achieving bidirectional conduction block (Figure 4A). We also confirmed the absence of conduction through the accessory pathway with nodal retrograde VA activation (Figure 4B).

The postinterventional period was uneventful and the patient was discharged 2 days later. At a 6‐month follow‐up, the patient was asymptomatic.

## 2 DISCUSSION

We report an unusual case of CTI dependent AFL with spontaneous 1:1 atrioventricular (AV) conduction and pronounced ST‐segment elevation in aVR, previously described as a “shark fin phenomenon” or “triangular QRS‐ST‐T wave,” mimicking a broad complex tachycardia with a ventricular origin. To the best of our knowledge, this is the first reported case of AFL with a 1:1 ventricular response (ventricular rate 250 bpm) associated with myocardial injury, as indicated by these specific ECG appearances and myocardial enzyme release.

Diffuse ST depression, with reciprocal ST elevation in aVR, can be a baseline secondary repolarization abnormality (eg, from LVH, LBBB), rate‐related (eg, tachyarrhythmia) or caused by metabolic/toxic causes (eg, hypokalemia, digoxin). Diffuse ST depression, with reciprocal ST elevation in aVR, can represent circumferential subendocardial ischemia from any shock state (eg, sepsis, gastrointestinal bleeding, pulmonary embolism, acute aortic dissection), and is a high‐risk finding. If caused by ACS, this can be from any acute coronary occlusion, or multi‐vessel disease, and can accompany other signs of acute coronary occlusion or be the only sign.

According to the current guidelines, ECG changes with ST elevation in aVR and diffuse ST depression (over 8 leads) are a strong indicator of global ischemia involving the left main stem (LMS) or affecting all three coronary vessels.

Having excluded coronary stenosis, possible mechanisms of induced ischemia may include coronary spasm or a mismatch of supply‐demand in the coronary bed, due to aortic stenosis or anemia. Coronary spasm, in this case, is less likely due to the global pattern of ischemia on the ECG and immediate regression of ST changes in lowering of the ventricular response, suggesting a direct link between high heart rate and deterioration of myocardial perfusion. Analyzing the parameters of such a process may be crucial in understanding the pathophysiology of tachycardiomyopathy. Calcium homeostasis has been proven to play a key role, affecting diastolic function and impairing excitation‐contraction coupling. Although disturbances of coronary flow during tachycardia have been demonstrated, there is still ongoing research regarding the mechanisms of initiation and maintenance of tachycardiomyopathy.

Myocardial ischemia may lead to sudden cardiac death (SCD) due to VF. Among supraventricular tachycardias, pre‐excitation with atrial fibrillation is the only situation shown to be associated with a substantial risk of SCD. In this case, the persistence of tachycardia and global ischemia could potentially have led to SCD. Post‐mortem diagnosis is often not possible, and typical flutter and supraventricular tachycardias with rapid ventricular response inducing serious myocardial injury may represent an under‐recognized cause of SCD.

Our case report demonstrates that, as well as well‐established causes of broad complex tachycardias such as hyperkalemia, supraventricular tachycardia (SVT) with AV conduction abnormalities and antidromic AVRT, the possibility of an ST abnormality mimicking a broad QRS morphology should be considered. Classic criteria (Brugada criteria, Vereckei algorithm) differentiating ventricular tachycardia from SVT presenting with a wide QRS and negative QRS concordance in the precordial leads, could be misleading. Syncope/presyncope has been reported in patients with AFL and 1:1 AV conduction. According to the recently published guidelines, incorrect diagnosis of such a sustained tachycardia as ventricular in combination with syncope or presyncope may lead to intracardiac defibrillator (ICD) Implantation for secondary prevention. Therefore, understanding the underlying mechanism is crucial for correct treatment.

## CONFLICT OF INTEREST

Authors declare no conflict of interests for this article.

